# Odontological Society of Pennsylvania

**Published:** 1891-11

**Authors:** 


### ODONTOLOGICAL SOCIETY OF PENNSYLVANIA.

THE regular monthly meeting of this Society was held April 4, 1891. In the absence of the essayist for the evening, various practical suggestions were offered and considered by the members present.

*Dr. Bassett.*—It may be remembered that a few months ago Dr. Bonwill had a cast presented of a case in which the temporary superior lateral was missing. It was interesting as being very rare. I saw a case the other day, and have a model of it, in which both superior laterals failed to erupt. I have brought the model here. It was a novelty to me, but I do not know how much it may be to the other gentlemen; I had never seen a case before, and thought it might be interesting to show it.

The case is that of a little girl about eight years of age. As shown, the sixth-year molars are erupted, but the other temporary teeth are not loosened.

By way of asking for information, I would like to know whether any of the gentlemen have had similar presentations, and whether such are common or not. We frequently see cases where one or both of the superior laterals of the permanent set are missing, but I never before saw one where they were missing in the temporary set.

*Dr. Truman.*—I have seen cases exactly like it. It is rarely we have irregularity in the deciduous teeth. I presume the teeth are developed properly in the jaw.

*Dr. Deane.*—You think those deciduous teeth would be forced out later on in life?

*Dr. Truman.*—I should think so, when the permanent series come.

*Dr. Rehfuss*—A short time ago I saw a case where two inferior centrals were united. The peculiarity about it was that they were exactly similar. They could not have been put together closer than they were. They were exactly mates. It was not my case, but that of Dr. Robinson. They looked like ivory. It was a beautiful specimen of temporary teeth. I do not suppose that it was exactly a novelty, but the position of the teeth being so regular made it a particularly interesting specimen.

*Dr. Truman.*—I suppose Dr. Rehfuss means the union of the teeth. I think most of the members here have seen cases of that kind; at least I have, quite a number of them,—deciduous teeth united in that way and also permanent teeth. I do not regard that as a very extraordinary thing, although it is not common. A great many persons may go through many years of practice and see nothing of the kind. Clinical work in colleges will show more than private practice.

*Dr. Bonwill.*—All I have to say in regard to this is merely to give my experience with two cases I have had after thirty-six years in practice; they both came in the same year. One where both laterals were absent, and one case where the right superior lateral was missing, and in the other the left superior lateral was not in place. I am watching this case with a great deal of interest. I suppose there will never be any permanent laterals in their place. It is rather singular that I should never meet with anything of that kind in thirty-six years, and both came about the same time,—the same week.

*Dr. Guilford.*—How old is this child?

*Answer.*—About eight years.

Dr. Long exhibited an artificial tooth with a metal pin. I think it is a great improvement on any of the others. On account of having a screw cut in the body of the tooth, you can file your post any shape you want to suit, and then take out your post and grind the tooth to fit the root; and you are not troubled with the post in the way; then put a little cement on the post and screw it in the tooth, and after the cement hardens you can place the tooth in position.

*Question.*—Is this the old-fashioned pivot-tooth?

*Answer.*—There is a screw cut in the tooth that the pin fits in. I think the advantage is in taking out the post and grinding the tooth to fit. They are made by Johnson & Lund. The thread is made when the tooth is moulded and biscuited, and then it is baked. When it is in the mould, the pin is screwed out, I suppose. It could not be baked in the mould.

*Dr. Rehfuss.*—That is the way it is done. I saw the specifications of the patent. They biscuit it and then bake it afterwards.

*Dr. Long.*—I present for examination some very fine thin steel for matrices. It comes from Johnson & Lund. They will sell any quantity, large or small. Ordinary shears will cut it.

Dr. Guilford spoke of shears gotten up by the S. S. White Company for cutting hard substances, and recommended them to the members present.

*Dr. Long.*—I use a pair of shears which I bought in a ten-cent store for cutting emery-cloth. The action is as satisfactory as a high-priced pair, and I would recommend them to any one who does not wish to spoil a good pair in such work.

*Dr. Guilford.*—I did not think of saying anything to-night until I was on my way here, and then it occurred to me that there was one subject that has concerned all of us for a long while, and yet one in which many of us have not been sufficiently interested. It is the erosion of the teeth. I do not mean the erosion that we generally call by that name, where the surfaces are polished and smooth. I mean that resulting from any cause whatever. You know from your own observation the bad effect of certain medicines upon the teeth. To avoid this, physicians have been in the habit of telling their patients to take certain medicines through a tube, but a person cannot put a tube in the mouth and pour liquids down the throat, as we would into a barrel through a funnel. The liquids have to be taken into the mouth, carried to the back part, then thrown forward against the teeth and again backward, before they can enter the stomach. The physicians have been blamed for doing a great deal of damage to the teeth by the administration of certain medicaments, but we cannot place all of the responsibility upon them. We find cases of erosion where the cause is so obscure that we cannot definitely trace it. We sometimes attribute it to the taking of phosphates, or of vegetable acids in the form of fruit, or to the action of acids formed in the oral cavity, assisted, perhaps, by the low order of vitality of the teeth at the time. For my own part, I have felt for a long while that the phosphates that are so freely used to-day are responsible for much injury to the teeth. I had a case not very long ago which seemed to confirm this theory very markedly. A friend of mine, who is a druggist in one of the suburbs of the city, has a small business and sells soda-water. I have had the care of his teeth for many years. His teeth are of good average quality, and so are the teeth of all his family. His health has been good all along; no change in his system that I know of. I saw his teeth two years ago, and they were then in fair condition. I saw him a few months ago, and I was very much shocked at their appearance. Two crowns were entirely destroyed, while others were badly eroded. On the approximal surfaces most of those in the anterior part of the mouth were entirely denuded of enamel, while on their labial surfaces the enamel was entirely gone from the upper half. I questioned him as to what he had been doing. I asked him if he had ever used phosphates. He told me that last summer he had used them almost exclusively for his beverage. He took what is called phosphate-soda. He had drunk a great deal of it. There is nothing else in his case that I could attribute that particular erosion to. I had another case showing the effect of other acids on the teeth of a lady who has been a patient of mine for a great many years. She came to me last spring, and her teeth were in good condition. Recently she called, saying that her superior anterior teeth were very tender at times. I looked them all over carefully, but there was not a cavity; the teeth seemed to have a natural appearance in every way. I finally took a napkin and wiped off the labial surfaces of the incisors. I noticed that when they were dry they had a crystallized appearance. Wherever the napkin touched the teeth they were exceedingly painful, while the touch of an instrument caused still greater suffering. There was so little wasting of the enamel that when the teeth were wet it did not show at all, as they retained their general form. I inquired into her case very closely, and finally ascertained that she had lived almost entirely on grapes during the past summer. We have seen the deleterious effect upon the teeth of grapes eaten in great quantities, especially in patients who have taken the grape-cure in Europe. Dr. Dixon mentioned to me the case of a young lady whom he had seen in the spring. She went to Europe, took the grape-cure, and came back in the fall with her teeth entirely honey-combed. In the case of my patient, I could attribute the harm done to no other cause but the acid of the grapes. I gave her proper directions, told her not to eat any more grapes, and prescribed the free use of prepared chalk around the necks of the teeth, especially before retiring.

The other case I had came to me a few days ago. It was that of a minister in this city, who is not only a close student, but has been very hard at work recently building a church. He is abstemious, does not smoke, and lives a regular and even life. His teeth were being eroded very much, as the druggist's had been. I inquired into his case, and could find nothing except that for a couple of years past he had been eating shaddocks for breakfast every morning.

We know the acid taste of this fruit, especially in the early spring. They are picked in a green state, and allowed to ripen on the way. I could attribute his difficulty to no other cause than that of eating this fruit. It seems to me that in all these cases the trouble was traceable to acids in some form.

There was another thing that attracted my attention. The erosion was confined to the right side. The centrals, laterals, and cuspids were particularly affected; the left were not so much, and it puzzled me to know why. It was not, of course, from any action of the brush, because he is a right-handed man, and it would show more on the left side if caused by mechanical action. I questioned him, and he said that for the last six months or more he had been eating upon the right side of his mouth, using the spoon in his right hand, and it probably came more in contact with the teeth on the right side than on the left. That seemed to me to be the reason why they were affected more on one side than on the other.

We have all been interested in this matter, and it seems to me that in all cases of this character we ought to try to get down to the facts, and find out, as nearly as we can, what has caused the harm, and some day we may be able to arrive at a reasonable conclusion in regard to it.

I should have mentioned another fact, and that is, so far as I know, we have never noticed any ill effects from eating grapes, except where the seeds are taken out. People who swallow the entire grape, without breaking the capsule in which the seeds are contained, suffer no injury to the teeth, but when the capsule is broken, the acid is liberated, and it is this that I believe to be the agent of harm in the matter.

*Dr. Deane.*—I would like to ask what the treatment would be in cases where the erosion is so marked.

*Dr. Guilford.*—I should try to get rid of the sensitiveness, and would forbid them using the article of diet suspected of being the cause. I said to the minister, continue your diet just as it has been, but stop eating shaddocks, and let me know in a month or so whether there is any improvement. In the second place, I recommend something to counteract the acid, generally precipitated chalk applied just before retiring.

I would like to ask if Dr. Truman noticed any effect of mineral waters upon the teeth when abroad.

*Dr. Truman.*—Not particularly. In regard to mineral waters, there is a great difference. Some are more or less acid; others are neutral; others are alkaline. I made some experiments in regard to that matter, and the rapidity with which the neutral mineral waters changed to acid, producing rapid development of micro-organisms out of the mouth, would indicate the probable effect on the teeth.

*Dr. Woodward.*—I have a very marked case of erosion, which I have observed for several years. The enamel and dentine appear to melt away from the gutta-percha, as well as from the amalgam and gold. The patient is in the habit of using acids for long periods to control the action of his liver, and maintains that this cannot be avoided. His teeth are almost destroyed. I think the erosion is caused by the acid, the dentifrice, and the brush. The acid condition softens the surface of the teeth slightly, which yields readily to the brush and polishing material. In ordinary cases the precipitated chalk will relieve the sensitiveness, especially if used liberally at night.

*Dr. Deane.*—Dr. Woodward brings my own case in mind. I am troubled with slow, sluggish action of the liver. I have to take a great deal of muriatic acid, and take it very strong. I take sometimes as high as twenty or twenty-five drops. I have not, as yet, felt any discomfort of the teeth, but I would like very well to find some substitute for the acid, as I am becoming uneasy as to the possible effect.

*Dr. Guilford.*—In Dr. Deane's case, I would say that the general condition of the system has something to do with the effect of acids upon the teeth. I have no doubt that many of us take as much acid as the minister did. He was in a run-down condition at the time; so much so, that it acted as a predisposition in his case. The lady I spoke of was also in a debilitated condition. I suppose that many of us are at times subjected to similar influences, but, owing to other conditions, we are not affected in the same manner. I do not think their teeth were especially affected because they had used more acids than others, but because of the then low condition of their systems. I have not paid any attention to temperament, only to the fact that they were overworked or overtaxed.

*Dr. Truman.*—I cannot quite agree with Dr. Guilford in the idea that temporary systemic conditions make the teeth more liable to destruction. I cannot see why that should be the case. The teeth remain unchanged, no matter what the general conditions of the organism may be. Therefore, whatever takes place must necessarily be external to the teeth, and not through any special and temporary loss of vitality.

In regard to the general subject of erosion, there is probably much yet to be learned. I have given my views frequently in the dental periodicals, and very recently in the INTERNATIONAL DENTAL JOURNAL, in the report of the New Jersey State Society, in which I went over the ground thoroughly, so that I have very little to offer in regard to it; but Dr. Guilford has brought up some points that were not then discussed, and to my mind they are quite important. This whole acid question—fruit acid—has not been sufficiently considered, for the reason, it seems to me, that dentists do not take a proper view of it. This subject of erosion by fruit acid has always been a matter of very great interest to me. I presume every one here will bear out the assertion I am about to make, that patients are more troublesome in the spring of the year, at the time we have our strawberry crop, than at any other period. I find that the acid of the strawberry known as the Wilson Seedling is very acid, and it brings about almost invariably a troublesome condition of the teeth, the sensitiveness being greatly increased.

The acid of lemons and fruits of similar character, I am inclined to think, is more destructive to teeth than any that our patients are in the habit of using. It is a very common thing for some persons to puncture lemons and take the juice directly into the mouth. Some persons like it, but I cannot witness it without a shudder.

The acid of lemons will destroy teeth in a very few weeks. I had an excellent illustration of that fact. In crossing the ocean, a member of my family, against my advice, made use of lemons to overcome sea-sickness. The teeth were all right when we left home, and in three weeks' time they exhibited marked signs of erosion on the surfaces of the anterior teeth. I have noticed this occurring constantly in the use of fruits of this character.

With regard to acid remedies much in use, I agree with Dr. Guilford. These phosphates are destroying teeth by the hundred. There is no question about it. The sooner the dental profession protests, the better. I think it is our duty to warn our patients.

I had a patient, a few years ago, that caused me the greatest possible trouble. She was suffering constantly with toothache. I examined the mouth over and over again. I gave her antacid washes, but the relief was only temporary. I could not discover the cause. I said to her, finally, "You are habitually in the use of some agent that is antagonizing my efforts." She did not know that she was. I finally pushed my inquiries to the tooth-powder she was using. Her answer was that she employed a powder recommended by a physician. She brought me some of it, and I went over the ingredients. I said, "Do you use tartaric acid in that powder?" "Oh, yes," she replied; "I was advised to use a little of it." The cause being discovered, the remedy was simple; the teeth yielded to treatment at once, and the cavities were filled properly.

Then there is the action of different mineral acids. I am surprised that our friend here, Dr. Deane, will use these in the way he does, and, though he may not feel any trouble now, he is bound to have it before long. Several years ago I had a patient employed in a confectionery establishment. Of course the confectioner's sweet always means an acid resulting. The destruction was almost universal and the sensitiveness beyond endurance. Before operating at all, I placed her under the effect of a daily wash of a solution of bicarbonate of soda; the result was very gratifying, and at the end of a week I was able to operate with comfort to her and myself.

I had another patient who was a worker in a chemical laboratory. She had beautiful teeth,—one of the most beautiful sets I think I ever saw for regularity and for density, which is to the dentist much more satisfactory than white teeth. She was not in the acid department, but still was so constantly affected by the atmosphere that it was impossible to save her teeth for any length of time. I finally advised her that, unless she could change her business, it would be impossible to save them.

On the whole, the matter of erosion I regard as altogether a chemical one; and to me it is immaterial whether it be the anterior or labial surfaces of the teeth that are worn away. It is unquestionably always due to the acid condition, and I do not, as I have stated in other papers and discussions, separate abrasion from erosion. Abrasion may be by simple attrition of one tooth upon another, and that attrition always advances, in my opinion, by an acid condition.

I had a very fine illustration of this some years ago. A prominent clergyman desired a tooth regulated. He had a beautiful set of teeth. The incisors were magnificent specimens. I placed on the regulating apparatus, and he did not appear for some time, to my annoyance. In about a month he returned, with the central incisor worn down a quarter of an inch from the cutting surface. I asked him what he had been doing to cause this disturbance. He replied, nothing. But I answered, "This single incisor could not have lost so much of its grinding surface without some irregular action." He then told me that the teeth annoyed him, and he had been biting the lower teeth against the central incisors. The abrasion was evident; but all the biting possible could not have worn it down to that extent in the course of four weeks without the aid of acid secretions. It was evident that the change produced at night from the neutral day secretion had worn down the tooth by the acid produced, and attrition had done the balance.

You know how rapidly the teeth of those who grind the teeth at night disappear on the cutting edge; how soon they are reduced to almost stumps. It is not the grinding solely, in my judgment, but a combination of acid and wear. I have always secured an acid reaction in mouths tested at night, though neutral in the daytime. If this be the case, we can understand erosion. As I said at the New Jersey State Society, I do not regard this action of erosion as a difficult problem; it is one of simple things; and if we look at it in the right light, always taking the acid view of the question from the beginning, it will lead us up to clear conclusions in regard to results.

*Dr. Tees.*—I find in my own practice among my regular patients who obey my instructions in the use of prepared chalk once or twice daily that they have no evil results from the use of acids. They may use fruit of any kind, lemons or anything else. In fact, in some cases I recommend the application of lemon juice around the gum. The most efficient antacid that I know of is prepared chalk.

I think the cases described by Dr. Guilford are exceptions. I do not suppose he meets on the average more than two or three a year, and I have no doubt such are in the mouths of those who never use the tooth-brush. I furnish prepared chalk to patients in half-pound packages, that they may have plenty of it. I have no doubt the use of it is a great benefit. No doubt we have all noticed how quickly the mouth responds to treatment when this is ordered. After the use of such preparations, the painful effect ceases, and we are able to operate with some degree of comfort.

*Dr. Chupein.*—I should like to ask Dr. Truman how he accounts for the highly polished condition of the erosion, if, as he says, it is due to chemical action.

*Dr. Truman.*—I will ask the gentleman to go back many years with me. Possibly he remembers the old clamps or clasps of some thirty years ago that used to be attached to the teeth. They were made of half-round wire, and to these were attached perhaps two or three teeth, and the invariable result of that was the wearing away of the teeth they were attached to, precisely as we see the erosion at the present time, and highly polished. I presume he will agree with me when I say that I have seen them cut half-way through the teeth. The irritation which was aroused by that half-round wire produced secondary depositions in the pulp, and the result was that the wire would go through the tooth without producing pain. Now, how was that effected? How was the polish produced? It was by the constant movement of the clasp and the movement of the secretions. You cannot get a polish by merely chemical action. You do not find any polish in teeth of a soft character where the acid acts with great rapidity. It is only in dense teeth. Secondly, this erosion occurs at middle life, when the teeth have nearly reached the maximum of density. This is my experience.

How do I account for the polish on the front teeth? There is a constant motion there of the muscles of the lip. In talking, of course, there is always motion of the teeth and lips. Then there is a constant flowing in and out of the secretions and the food constantly pressing on these surfaces. The tooth-brush may be a factor also, but it seems to me not a powerful one.

I hold that the polish is simply produced in that way, and I see no reason for any other explanation. I know it is a difficult thing to test, and we are obliged to reason from analogy, hence I brought up the clasp as an illustration, because I know Dr. Chupein and others here as old as myself remember the condition of things as a result of its use.

*Dr. Tees.*—Dr. Truman stated that cases of erosion were nearly always at middle life. I have seen a great many in the twenties. Erosion where a great deal of the tooth substance is worn away. The polish was there also.

*Dr. Truman.*—You may have dense teeth at twenty years of age. It is always confined to dense teeth.

*Dr. Chupein.*—I have never noticed the conditions except in middle life in very dense teeth.

*Dr. Tees.*—I had a patient to-day with two very distinct small cavities on the labial surfaces of the superior right canine and superior right lateral, a sixteenth of an inch deep, and probably the width of a small pin-head. I would like Dr. Truman to give his opinion of these two cavities. They were on the labial surfaces of the superior right canine and superior right lateral, two distinct deep cavities. The rest of the enamel was perfect. Now, what could have caused this?

*Dr. Truman.*—These were ordinary cavities?

*Dr. Tees.*—Yes; but why should those little, distinct cavities be there? Why should not the whole surface there have decayed? It has been within a year that the cavities have formed.

*Dr. Truman.*—The only explanation is that they always begin at a small point. There is one portion of the tooth which comes nearer the lip than any other point, and, as Dr. Kirk has demonstrated the glands of the lip to be always acid, it will then be understood why it would begin at a particular part on that surface.

*Dr. Tees.*—Dr. Flagg has done what Dr. Truman gives Dr. Kirk credit for.

*Dr. Truman.*—I must take exception to that. No one, as far as I am aware, has demonstrated facts as Dr. Kirk presented them. We know very well, in a general way, that the mucous glands are considered to exude an acid secretion; but you cannot always get an acid reaction from other portions of the mouth. Dr. Kirk was induced to make these experiments by the facts which I gave in regard to the secretions at night being powerful and more destructive at that time than during the day or active period. I took the position that the upper lip held the secretions against the teeth, and fermentation took place. Now, Dr. Kirk's demonstration was to prove that they were acid during the whole twenty-four hours, and he went into a series of very delicate examinations to demonstrate that fact. I do not know that it ever was done before. I therefore give him the credit.

*Dr. Tees.*—Dr. Flagg said that the mucous secretions of the mouth were acid. If the whole mucous secretions of the mouth are acid, of course the lips ought to be also; and he also explained that in a healthy condition of the mouth the secretions of the salivary glands were alkaline, and that the secretions of the mucous membranes, being acid, neutralized each other.

*Dr. Guilford.*—In regard to the matter of erosion, I have seen many varieties of it; the most common one, of course, being that of the disappearance of the enamel. I have seen it between the teeth and not upon the labial surfaces. I have also seen it in a sort of worm-shape distribution around the incisors and palatine surfaces; but the most singular case was that of a young lady, twenty-five or thirty years of age, whom I was called in to see. The fillings, which had been put in a few years before, were all perfect, but the whole surfaces of the enamel had dissolved, just as though the teeth had been made of candy and had been dipped in water several times. All of the fillings stood slightly above the surrounding tooth tissue. It was the only case of the kind that I have ever seen.

Subject passed.

*Dr. Deane.*—I have the subject here of statistics of manufacturers. I would like to ask for some instructions. It seems the agent is canvassing the matter in a new way. It seems to me the statistics would be of benefit to dentists. The agent has called on me and explained it so that it seems not to lessen professional dignity to fill out these blanks. I would like to know whether the Society objects to a member making the desired record. I have been asked this question by several members.

*Dr. Guilford.*—I think the agents have been asking too much. Certainly what they did ask for seemed to be obnoxious to those who were approached.

*Dr. Deane.*—This agent simply requires the number of plates that you have made for the year 1889, not bridge- or crown-work, or anything of that kind, as to the quantity of plates and how much they were worth, not individually but collectively. If you do not manufacture plates they do not want to know anything about it.

*Dr. Guilford.*—The Baltimore dentists were up in arms about it. They considered it inquisitorial. They would not give the facts wanted.

*Dr. Tees.*—I move that the resolution on the books be reconsidered.

*Dr. Rehfuss.*—I should be thoroughly opposed to rescinding this resolution. First, because this Society and the dentists of Baltimore have taken a stand in showing their position to the dental profession. The government has made a mistake in issuing this form,—that they acknowledge,—and this blank is a modification of the blank presented before. It has not the slightest thing in it in regard to the separation of mechanical from operative dentists. I do not think the plan would give correct statistics. I think the Society should allow the resolution to stand.

*Dr. Deane.*—I cannot quite agree with Dr. Rehfuss. I think he overlooked the point that, if it is possible to find out how many plates are made once in ten years, it would be of great value to the dentists, and that is all they ask for. They do not require you to say anything about your fillings, nor anything about the number of operations, but merely the number of plates manufactured; and they want to know, once in a stated period, how many plates are manufactured in the United States, and how much money was put into it. It seems to me that in thirty or forty years from now the statistics will be of great value to dentists.

*Dr. Truman.*—I think this Society or some other Society ought to take action to get the tariff taken off English teeth. It is impossible to get English teeth now without a great deal of trouble. The tariff was raised from 20 to 55 per cent. ad valorem.

*Dr. Guilford.*—I think I saw a statement of some one in the West who said that there was a mistake, and that they ought to have been rated under the name of manufactured articles, and not porcelain.

*Dr. Truman.*—I sent to Washington to our representative to get the facts, and he sent me a work covering everything. It gives a list of the old terms, and then says, "Refer to porcelain." Referring to porcelain, it has nothing to say with regard to teeth, but states that all porcelain not ornamented shall be at the rate of 55 per cent. ad valorem. If an attempt be made to import teeth, it will be found that the duty will be 55 per cent., and that is practically prohibitory.

